# Identification and Analysis of Immunodominant Antigens for ELISA-Based Detection of *Theileria annulata*

**DOI:** 10.1371/journal.pone.0156645

**Published:** 2016-06-07

**Authors:** Huseyin Bilgin Bilgic, Tulin Karagenc, Serkan Bakırcı, Brian Shiels, Andrew Tait, Jane Kinnaird, Hasan Eren, William Weir

**Affiliations:** 1 Faculty of Veterinary Medicine, Department of Parasitology, Adnan Menderes University, Isıklı Mevki, 09016, Aydın, Turkey; 2 Institute of Biodiversity Animal Health and Comparative Medicine, College of Medical, Veterinary and Life Sciences, University of Glasgow, Bearsden Road, Glasgow, G61 1QH, United Kingdom; Institut national de la santé et de la recherche médicale—Institut Cochin, FRANCE

## Abstract

Tropical or Mediterranean theileriosis, caused by the protozoan parasite *Theileria annulata*, remains an economically important bovine disease in North Africa, Southern Europe, India, the Middle East and Asia. The disease affects mainly exotic cattle and imposes serious constraints upon livestock production and breed improvement programmes. While microscopic and molecular methods exist which are capable of detecting *T*. *annulata* during acute infection, the identification of animals in the carrier state is more challenging. Serological tests, which detect antibodies that react against parasite-encoded antigens, should ideally have the potential to identify carrier animals with very high levels of sensitivity and specificity. However, assays developed to date have suffered from a lack of sensitivity and/or specificity and it is, therefore, necessary to identify novel parasite antigens, which can be developed for this purpose. In the present study, genes encoding predicted antigens were bioinformatically identified in the *T*. *annulata* genome. These proteins, together with a panel of previously described antigens, were assessed by western blot analysis for immunoreactivity, and this revealed that four novel candidates and five previously described antigens were recognised by immune bovine serum. Using a combination of immunoprecipitation and mass spectrophotometric analysis, an immunodominant protein (encoded by *TA15705*) was identified as Ta9, a previously defined T cell antigen. Western blotting revealed another of the five proteins in the Ta9 family, TA15710, also to be an immunodominant protein. However, validation by Enzyme-Linked Immunosorbent Assay indicated that due to either allelic polymorphism or differential immune responses of individual hosts, none of the novel candidates can be considered ideal for routine detection of *T*. *annulata*-infected/carrier animals.

## Introduction

Tropical or Mediterranean theileriosis is an economically important bovine disease which is widespread between longitude 30°W—150°E and latitude 15°- 60°N [1]. The disease is caused by the protozoan parasite *Theileria annulata* and is transmitted by several species of ixodid ticks of the genus *Hyalomma* [2]. The disease imposes serious constraints upon livestock production in developing countries. In cattle that survive acute disease, a long-lasting carrier state develops, a condition that is associated with significant production and economic losses [3,4]. The carrier state is characterised by the presence of low numbers of piroplasm-infected erythrocytes [5] that are infective for feeding tick larvae and/or nymphs and thus the carrier state is important for the transmission of the parasite. Identification of carrier animals is crucial for an accurate assessment of disease epidemiology in endemic areas in order that effective control strategies may be designed and, therefore, a high throughput sensitive diagnostic assay is required.

Diagnosis of *T*. *annulata* infection in cattle is mainly based on microscopy, molecular methods or serological assays and each has its own advantages and disadvantages. Microscopic examination based on detection of macroschizont-infected leukocytes in Giemsa-stained lymph node biopsies or piroplasms in Giemsa-stained peripheral blood smears is effective for diagnosis of acute cases. However, microscopically, it is difficult to discriminate *T*. *annulata* from non-pathogenic *Theileria* spp and these approaches lack the sensitivity to detect carrier animals with low piroplasm parasitaemia. Molecular methods such as the polymerase chain reaction (PCR), reverse line blotting (RLB) or loop-mediated isothermal amplification (LAMP) can be used as sensitive and specific tools for specific detection of *T*. *annulata* DNA in both the tick vector [6,7] and the bovine host [8–15]. However, issues such as low levels of parasite DNA in carrier animals [14] and competition between primers for species-specific conserved regions of certain genes such as 18S ssrRNA [16,17] serve to reduce test sensitivity: thus herd infection rates may be underestimated [16,18,19]. Additionally, for large-scale field studies molecular methods have the disadvantages of being expensive, labour intensive and require a degree of technical expertise [9,10,20].

Serological tests remain the most appropriate assays for large-scale studies aimed at identifying carrier animals and determining the distribution of the disease [20,21]. These rely on the fact that the bovine immune system is exposed to a variety of *T*. *annulata*-encoded antigens in the course of infection [22] and thus antibodies that react with different life-cycle stages of the parasite are generated. Immune serum has been shown to block invasion of sporozoites *in vitro* [23,24] and monoclonal antibodies can neutralise sporozoites and ablate infectivity [25,26]. Antibodies to free merozoites have been demonstrated *in vivo* following primary infection [27], however, no reactivity has been detected against infected erythrocytes [28] or the surface of the infected leucocyte [29].

Due to significantly elevated antibody titres during the later phase of infection, enzyme-linked immunosorbent assays (ELISA) are considered to be suitable for the detection of *T*. *annulata* carrier animals [20]. ELISA has several advantages over other assays such as the immunofluorescence antibody test (IFAT), including the ability to test large numbers of samples easily, rapidly and economically and can display greater sensitivity and specificity. Consequently, ELISA has become an effective tool for large-scale epidemiological studies in endemic regions [20,30]. Different forms of antigen preparation such as crude antigen extracts and stage-specific recombinant antigens have been used to develop ELISAs. However, ELISAs with crude antigen extracts, obtained from macroschizont and piroplasms [31,32], provided lower sensitivity and specificity than IFAT. Moreover, there were issues with shelf-life and quality control. A limited number of stage-specific recombinant antigens from the sporozoite (SPAG), schizont (NC10, TaD and TaSE) and merozoite/piroplasm (Tamr-1) stages have previously been identified and tested [33–35]. However, tests using TaD, TaSE [36], SPAG [22] and NC10 [33] were compromised by poor sensitivity as very low levels of reactive antibody are generated during infection, while TaD [36] and Tamr–1 [33] showed an insufficient level of specificity. Conserved proteins such as heat-shock 70 have been used as the basis for an indirect ELISA for *Theileria* sp. (China) in small ruminants [37]. However, despite recombinant TamHSP70 protein [38] sharing 98% similarity, at the nucleotide level, with one of the predicted HSP70 genes of *T*. *annulata*, TA14920, no serological response against it could be detected [36].

Over the past decade, the merozoite/piroplasm surface antigen Tams and schizont surface protein TaSP have been defined as immunodominant surface proteins [39–41]. ELISAs have been developed [20,30,42–44] but, these have been shown to have issues with sensitivity and specificity [20,42,45] and both antigens possess a high level of polymorphism within and between isolates [46–49].

It is evident that ELISA assays offer the potential to detect *T*. *annulata* with a high level of sensitivity, if suitable immunodominant target antigens can be identified. Given that serum from infected animals reacts strongly with crude *T*. *annulata* infected cell extracts [33], such antigens potentially exist, and if they can be identified, should be evaluated in a diagnostic ELISA. In recent years, bioinformatic tools have become available to facilitate the mining of genomic sequence databases and may aid in identification of candidate antigens [50,51]. Candidate genes may then be cloned and expressed as recombinant protein to evaluate their immunogenicity [52–54]. A number of different properties of predicted proteins may be used in an attempt to identify putative antigens. For example, the ratio of non-synonymous to synonymous substitutions (d_N_/d_S_) between orthologues of closely related *Theileria* species was shown to be elevated in genes encoding proteins predicted to be expressed on the surface of the merozoite stage [54], indicating they may be under positive diversifying selection. Thus, stage-specifically expressed polypeptides possessing membrane retention motifs such as signal peptides (SP), glycosylphosphatidylinositol (GPI)–anchor and/or transmembrane domains (TMD) with elevated d_N_/d_S_ values may represent antigens recognised by a protective acquired immune response and be immunodominant.

Given that the *T*. *annulata* genome encodes in excess of 3,500 genes, it is reasonable to assume that undiscovered immunodominant antigens exist and thus represent potentially superior targets for an improved ELISA test. In the present study, we investigated whether additional immunodominant antigens do indeed exist and tested identified candidates as suitable targets for an ELISA. We successfully identified a number of novel parasite-encoded antigens, however none of these proved suitable for the development of an improved ELISA.

## Materials and Methods

### 1. Parasite material and serum samples

A panel of schizont-infected cell lines representing isolates from a number of countries was used to prepare protein extracts and is listed in Table A in S1 File. Reference control anti-serum samples were generated in experimental infections conducted previously [33]. Briefly, in order to more closely represent field conditions where parasite diversity is high, animals were infected using *T*. *annulata* / Ankara sporozoite stabilate at 0.2 tick equivalent (t.e.) dose and then challenged with a heterologous sporozoite stabilate, *T*. *annulata /* Gharb, at 1 t.e. The initial dose was sub-lethal and sufficient to stimulate immunity, while the secondary dose was more substantial and would have been lethal to naïve cattle. Serum samples were obtained from blood taken in plain vacutainer tubes at the following time-points: two hours before infection; days 7, 14, 21, 28 and then 28-day intervals until challenge; one day before challenge; days 7, 14, 28 and 42 following challenge. Additionally, a total of 355 serum samples were collected from cattle with or without a history of theileriosis around Aydın, Izmir and Manisa provinces of Turkey, where tropical theileriosis is endemic.

### 2. Identification of candidate antigens

Genes encoding putative immunodominant antigens were bioinformatically identified using the *T*. *annulata* genomic sequence resource (http://www.genedb.org) based on the following criteria: possession of a signal peptide (SP), one or more transmembrane domains (TMD) or a glycosylphosphatidylinositol (GPI) anchor, an elevated ratio of non-synonymous to synonymous substitutions (d_N_/d_S_) between orthologous genes in the *T*. *annulata* and *T*. *parva* genomes. Expressed sequence tag (EST) data was also included to evaluate stage-specific expression of each candidate gene. d_N_/d_S_ values and EST data of candidate proteins were previously generated [54].

### 3. Cloning, expression, and purification of recombinant proteins

Bioinformatically identified candidate genes, were cloned using the Gateway® (Invitrogen™) *in vitro* recombination method [55] and expressed as recombinant protein. Plasmids containing each protein-encoding gene fragment were purified using a plasmid purification kit (QIAGEN™), then sequenced using a commercial service (MWG Biotech). After confirmation of the presence of the correct insert, pDEST17 recombinant plasmids were transformed into *E*. *coli* strain BL21(AI) and used for protein expression, following the supplier’s protocol. Recombinant proteins were purified and eluted under denaturing conditions using Ni-NTA columns. The eluate of each recombinant protein was electrophoresed on 12% SDS-PAGE for 2 hours at 100 V. Following electrophoresis, gels were stained with 0.5% Coomassie Brilliant Blue G-250 (Sigma) in distilled water. The molecular mass of each recombinant protein was estimated relative to a set of molecular markers (6–175 kDa, BioLab). The protein concentration of each fraction was determined by the Bradford method (Bio-Rad, Munich, Germany). Recognition of recombinant proteins by antibody was evaluated by western blotting using reference anti-sera from immune animals. Reactivity was compared to that displayed by previously identified recombinant antigens, expressed and purified using standard procedures (see Table B in S1 File).

### 4. Preparation of protein extracts and SDS-polyacrylamide gel electrophoresis (PAGE)

Cryopreserved macroschizont-infected and uninfected (BL20) cell line stabilates (see Table A in S1 File) were thawed and cultured as described previously [56]. Cell pellets with approximately 10^7^ cells were used to prepare protein extracts as described by Ilhan [25] and stored at -20°C. SDS-PAGE was carried out as previously described [57], using the Biorad mini or midi Protean II gel electrophoresis systems. The acrylamide concentration of resolving gels varied from 10% to 12%.

### 5. Western blot analysis with immune serum

Immunodominant protein bands in cell lysates and reactivity of recombinant proteins were identified by western blotting. Standardisation of protein concentrations, serum and conjugate dilutions was carried out by serial dilution of each recombinant protein and protein extracts of different isolates. For western blot analysis, equal concentrations of protein extracts and/or 100 ng of recombinant proteins were loaded into each lane with SDS-PAGE loading buffer. Immunoblotting of polyacrylamide gels was adapted from Towbin, [58]. Proteins were transferred onto nitrocellulose filters (Hybond C, Amersham) in transfer buffer using a Bio-Rad Trans blot cell wet system (25 mM Tris base, 192 mM glycine, 20% methanol) at 300 mA for 1 hour. The filters were blocked with Tris-saline buffer containing 5% non-fat dried milk (Marvel) and all washing steps were performed using Tris-saline buffer (10 mM Tris.HCl, pH 7.4, 150 mM NaCl, 0.1% Tween 20). A panel of immune bovine sera was used to evaluate immunodominant protein bands in cell lysates and reactivity of recombinant proteins. This comprised serum samples from day 0 (pre-immune) and day 28, 84 and 140 post-primary infection and day 7, 28 and 42 after secondary challenge. Bovine sera were diluted at 1:500 and alkaline phosphatase-conjugated secondary antibody (anti-bovine IgG whole molecule, Sigma, A-0705) was diluted at 1:20,000 in blocking buffer. Washed filters were then developed by incubation with 5-bromo-4-chloro-3-indolyl-phosphate and nitroblue tetrazolium (Kirkegaard and Perry Laboratories Inc.). The reaction was stopped and bands were detected after washing the filters in tap water. Reactivity of each recombinant protein was determined based on visual analysis of band intensity and recorded as none (-), very low (+/-), low (+), medium (++), high (+++) or very high (++++).

### 6. Immunoabsorption of antibodies in immune serum and blocking detection of antigens in infected cell lysates

An immunoabsorption assay was carried out to determine whether immunogenic recombinant proteins represented any of the immunodominant bands detected by immune serum in a *T*. *annulata*/Ankara D7 infected cloned cell line lysate. Antibodies against each immunogenic recombinant protein in post-challenge day 42 immune serum were removed by modifying a previously published method [59]. 200 μg of each recombinant protein was used to absorb reactive antibodies from immune serum separately. As a control, 10 ml of immune serum was incubated under identical conditions but minus recombinant protein. Absorbed serum samples were then used in parallel western blot analysis of *T*. *annulata*/D7 cell extracts together with each matching recombinant protein.

### 7. Immunoprecipitation of infected cell lysate and mass spectrophotometric analysis

To identify the immunodominant bands detected by immune sera in cell lysates of the *T*. *annulata*/Ankara D7 clonal cell line, immunoprecipitation (IP) was performed using Dynabeads M-280 Tosylactivated (Dynal^®^ Biotech) and purified IgG fractions. IgG was purified from post-challenge day 42 immune sera using Pierce^®^ Thiophilic Adsorbant (Thermo Scientific) following the manufacturer’s protocol. After eluting IgGs, protein concentration was determined using the Bradford method. Eluate with an IgG concentration of >300 μg was then precipitated with ProteoExtract^®^ Protein Precipitation Kit (Calbiochem) following the manufacturer’s instructions and resuspended in 400 μl of 0.1 M borate buffer, pH 9.5. Then, 200 μl of ready-to-use Dynabeads (Dynabeads M-280 Tosylactivated, Dynal^®^ Biotech) were coated with 400 μl of purified IgG. 1.5 ml of cell lysate prepared from 10^7^ *T*. *annulata*/Ankara D7 infected cells was mixed with 25 μl of coated beads (10^8^ beads) following the supplier’s protocol. To elute precipitated proteins, 50 μl of 1 x SDS sample buffer was added and the beads boiled for 5 minutes. The tube was then placed on a magnet for 2 minutes and the supernatant containing target immunoprecipitated proteins transferred to a fresh tube and stored at -20°C until required. Samples were then analysed on two 10% SDS-PAGE gels in parallel. After electrophoresis, one of the gels was used for western blotting using immune bovine reference serum (1:500) as primary antibody, while the second gel was fixed and stained using 0.5% Coomassie Brilliant Blue G-250 (Sigma). The locations of dominant bands in the stained gels were determined relative to the dominant bands detected on the western blot. Five bands were then cut from the stained SDS-PAGE and sent for sequencing. The peptide sequence of each band was determined by LC-tandem mass spectrophotometric analysis at Glasgow Polyomics, University of Glasgow. The resulting data was then analysed using MASCOT [60].

### 8. Indirect ELISA and IFAT

Indirect ELISAs were established using recombinant versions of two immunodominant proteins, TA15705 and TA15710, in order to test diagnostic efficacy in comparison to IFAT using a panel of sera collected from cattle in the field, with or without a history of theileriosis. An indirect ELISA was also established using TaSP protein to provide comparison to a previously validated ELISA. Indirect ELISAs were carried out as described by [33]: 96 well polystyrene ELISA plates (Dynatech, Immulon, Type I) were coated with 1 μg recombinant protein; washing was performed using PBS buffer with 0.25% Tween20 (Sigma) and PBS/0.5% Tween 20 containing 5% skimmed milk powder (Marvel) was used for blocking. Absorbance at 450 nm was measured using an ELISA reader (ELX 808, Biotech). Optimal differentiation between OD readings of positive (C++) and negative sera (C-) was obtained using different titration methods. Antigen, serum and conjugate dilutions of ELISAs with different recombinant proteins are listed in Table C in S1 File. Optimal ELISA cut-off values were chosen by two-graph response operating characteristic curves (TG-ROC) [61]. Positive and negative threshold percentage positivity (PP) values were chosen arbitrarily to obtain the optimal sensitivity and specificity using known positive (n = 200 experimentally infected animals) and negative (n = 180 uninfected controls) serum samples. Cross-reactivity with serum samples derived from experimental infections with *Theileria parva*, *T*. *buffeli*, *T*. *orientalis*, *Babesia bovis*, *B*. *bigemina*, *Anaplasma marginale* and *Trypanosoma evansi* was also tested.

The diagnostic efficacy of indirect ELISAs was tested in comparison to IFAT as a gold standard. IFAT, based on the method of [62], was performed using *T*. *annulata* macroschizont-infected mononuclear cells as antigen. Each antigen slide included the following: 10 μl of the serum at a dilution of 1:80 in PBS, *T*. *annulata* positive and negative control serum samples diluted at 1:160 in PBS and PBS alone as a conjugate control. Rabbit anti-bovine IgG conjugated to fluorescein isothiocyanate (FITC) (Sigma) at a dilution of 1:160 in PBS was used as secondary antibody. Slides were examined using an Olympus BX51 fluorescence microscope. The degree of fluorescence was recorded as strong (+), weak (+/-) or no fluorescence (-). Only strong fluorescence (+) was accepted as being positive. An IFAT titre of 1:80 was taken as the cut-off value for the positivity of serum samples.

### 9. Sequence polymorphism of immunodominant antigens among isolates

Protein-encoding gene fragments of immunodominant antigens derived from different isolates of *T*. *annulata* were cloned into pDONR221 plasmid using the Gateway® (Invitrogen™) *in vitro* recombination method [55], following the supplier’s protocol. Recombinant plasmids were purified using a plasmid purification kit (QIAGEN™) and the respective inserts sequenced by a commercial service (MWG Biotech).

### 10. Ethical aspects

This study was approved by Adnan Menderes University Animal Experiment Ethic Committee dated 7/2/2006 in accordance with decision number B.30.2.ADÜ.0.06.00.00/124-HEK/2006/0022.

## Results

### 1. Bioinformatic properties of identified candidate proteins

Seven top-ranking candidate antigen genes (*TA06510*, *TA20440*, *TA13755*, *TA15040*, *TA08425*, *TA20980* and *TA11610*) were bioinformatically identified based on possessing one or more of the following: SP, TMD/GPI, and elevated d_N_/d_S_ ratio. EST data indicated that TA06510 is only expressed by the macroschizont stage, and that all other candidates were expressed in both macroschizont and merozoite stages. In addition, *TA11610* (HSP70) was indicated as being expressed by the piroplasm (Table 1). Of these seven candidates, *TA08425* and *TA20980* were found to be identical to two previously identified antigen-encoding genes, namely *Tamr-1* [63] and *NC1* [33]. For *TA08425* and *TA20980*, initial results indicated a lack of specificity and/or sensitivity (data not shown) and they were not taken forward and expressed as recombinant proteins. In addition, attempts to clone *TA15040* were unsuccessful. The remaining candidates, *TA06510*, *TA20440*, *TA13755*, *TA11610*, together with schizont surface protein TaSP, were cloned and expressed as recombinant proteins. Protein bands of the predicted molecular mass were observed for each recombinant protein, with the exception of recombinant TaSP. Recombinant TaSP showed a larger molecular mass (47 kDa) than predicted (19.9 kDa) and as suggested previously this is likely due to conformational expansion within PQ-rich regions [40,64]. For comparison against other previously identified recombinant antigens across different stages, fusion proteins representing Tams1, SPAG, Mero1, TA03155, TA10720, TA06470, TA16025 and TA17485 were expressed from pre-existing constructs at the University of Glasgow. Properties of each of these proteins are given in Table 1.

**Table 1:** Properties of bioinformatically identified candidate antigens and previously identified antigens

T.annulata_id	T.parva_id	dN	dS	dN/dS value	Protein identity	Nucleotide identity	Macro stage	Mero stage	Piro. stage	chr. location	Product	SignalP	TMHMM	GPI anchor	NLS	Protein info. (putative)	Conserved for *Theileria*	^f^Motif	^f^Domain info. (number)	Gene ontology (molecular function)	Apicoplast targeting	Rhoptry/microtubules	Macro EST hits	Mero EST hits	Piro EST hits	Mass (kDa)	Immunogenicity
TA06510*^a^	TP01_0939	0.23	1.43	0.16	63.5	72.9	1	0	0	1	hypothetial protein	√	1	√	-	-	-	-	F(1)	-	-	-	11	0	0	37	++
TA20440*^a^	TP01_0522	0.31	0.76	0.40	57.1	72.6	1	1	0	1	hypothetical membrane protein (TaSP alike)	√	3	-	-	-	-	-	F(1)	-	√	-	2	6	0	28	⫠
TA13755*^a^	TP02_0543	0.29	0.88	0.32	59.6	71.3	1	1	0	2	hypothetical prot. (TaSP alike)	√	3	-	-	-	-	-	F(1)	-	√	-	6	2	0	53.5	⫠
TA15040*	TP02_0777	0.22	1.02	0.21	69.2	74.6	1	0	0	2	hypothetical prot., conserved	√	3	-	-	-	-	-	F(1),DH	Zinc ion binding	√	-	20	0	0	25.5	nd
TA15705^a^^,^^c^	TP02_0895	0.33	1.05	0.32	58.9	68.7	1	0	0	2	hypothetical protein (Ta9)	√	-	-	-	secreted	-	-	-	-	-	-	10	0	0	35	++++
TA15710^a^^,^^d^	TP02_0896	0.47	1.34	0.35	47.4	63.0	1	0	0	2	hypothetical protein	√	-	-	-	secreted	-	-	-	-	-	-	4	0	0	38	++++
TA15685^a^^,^^d^	TP02_0890	0.34	0.89	0.38	54.8	70.4	0	0	0	2	hypo. protein	√	-	-	-	secreted	-	-	F(1)	-	-	-	0	0	0	28.1	+++
TA15690^a^^,^^d^	-	-	-	-	-	-	1	0	0	2	hypo. protein	√	-	-	-	secreted	-	-	-	-	-	-	10	0	0	35.9	+++
TA15695^a^^,^^d^	TP02_0888	0.77	20.9	0.04	29.5	51.4	1	0	0	2	hypo. protein	√	-	-	-	secreted	-	-	-	-	-	-	17	0	0	18.8	++
TA11610*^a^	TP02_0148	0.01	0.85	0.01	97.8	88.0	1	1	1	2	heat shock 70 protein	-	-	-	1	-	-	-	-	ATP binding	-	-	13	31	25	71	⫠
TA17315^a^^,^^b^	TP04_0051	0.31	1.28	0.24	59.9	68.5	1	1	0	4	surface protein precursor (TaSP)	√	3	-	-	integral membrane	-	P	F(1)	Copper ion memb. transp	-	-	11	7	0	35.5	++++
TA17050^b^^,^^e^	TP01_1056	0.2	0.72	0.28	72.9	77.4	0	1	1	1	mero/piro surf. antigen (Tams)	√	1	√	-	mero. antigen	-	-	F(1)	-	-	-	0	10	12	32.3	+++
TA03755^b^^,e^	TP03_0287	0.43	1.32	0.33	49.9	63.6	0	0	0	3	sporozoite surface antigen (SPAG)	√	1	√	-	sporo. p67 surf. antigen	-	-	-	-	-	-	0	0	0	93	⫠
TA08425^b^^,^^e^	TP04_0437	0.34	1.15	0.29	58.2	67.6	1	1	0	4	T.parva microneme-rhoptry antigen (Tamr-1)	√	1	√	1	-	1	-	F(4)	-	-	1	1	2	0	101.9	nd
TA13810^e^	TP02_0551	0.09	0.59	0.16	83.4	84.6	0	1	1	2	ts-chitose type piroplasm surf-like protein (MeroI)	√	1	√	-	-	-	-	F(1)	-	-	-	0	9	2	26.8	+
TA20980^a^	TP01_0380	0.44	2.81	0.16	51.2	62.2	1	1	0	1	proline-rich hypo. prot. (NC1)	√	-	-	1	-	-	-	-	-	-	-	2	4	0	111.1	nd
TA16090^a^	TP01_0966	0.42	1.57	0.26	51.8	63.9	1	0	0	1	glutenin, putative (NC10)	-	-	-	-	-	-	-	-	-	-	-	6	0	0	119.5	nd
TA03155^e^	-	-	-	-	-	-	0	0	0	1	Tash1-like protein	√	1	-	1	Tash AT-hook prot.	-	P, D	-	-	-	-	0	0	0	35.7	⫠
TA10720^e^	TP04_0646	0.06	0.91	0.07	87.6	84.8	0	1	1	4	HSP90 homologue	√	1	-	-		-	-	H	ATP binding	√	-	0	2	1	104.2	-
TA06470^e^	TP01_0934	0.11	2.61	0.04	82.3	76.5	0	1	1	1	chaperone prot. (HSP90 homologue)	√	-	-	1		-	P	H	ATP binding	-	-	0	1	7	115.7	-
TA16025^e^	TP02_0955	0.47	1.16	0.40	43.2	65.6	1	0	0	2	PQ-rich, SVSP^f^	√	-	-	-	Tash AT-hook prot.	-	P, T	-	-	-	-	4	0	0	64.5	-
TA17485^e^	TP01_1225	0.48	1.53	0.31	44.9	62.4	1	1	0	1	SVSP^f^	√	-	-	-		1	-	F(1)	-	-	-	1	1	0	47.5	-

* bioinformatically identified genes encoding candidate antigenic proteins in the *T*. *annulata* genome sequence based on possession of SignalP, GPI anchor, TMHMM, dN/dS values and EST data.

^a^ genes which were cloned and expressed as recombinant proteins to evaluate immunogenic properties.

^b^ previously identified antigens and genes encoding antigenic proteins presumed to be immunogenic.

^c^ gene encoding protein which was identified by immunoprecipitation and mass spectrophotometric analysis.

^d^ *TA15705* (Ta9) paralogue family proteins.

^e^ recombinant proteins expressed from available constructs.

^f^ used to indicate the abbreviations used for the FAINT (F), DHHC-type (DH), Histidine kinase-like ATPases (H) domains and PEST (P), DNA binding (D), TasHN (T) motifs.

^nd^ immunogenic property of the proteins identified in previous studies.

*T*.*annulata*_id and *T*.*parva*_id; indicates GeneDB (http://www.genedb.org) accession numbers of each gene.

### 2. Reactivity of recombinant proteins

**Fig 1:**
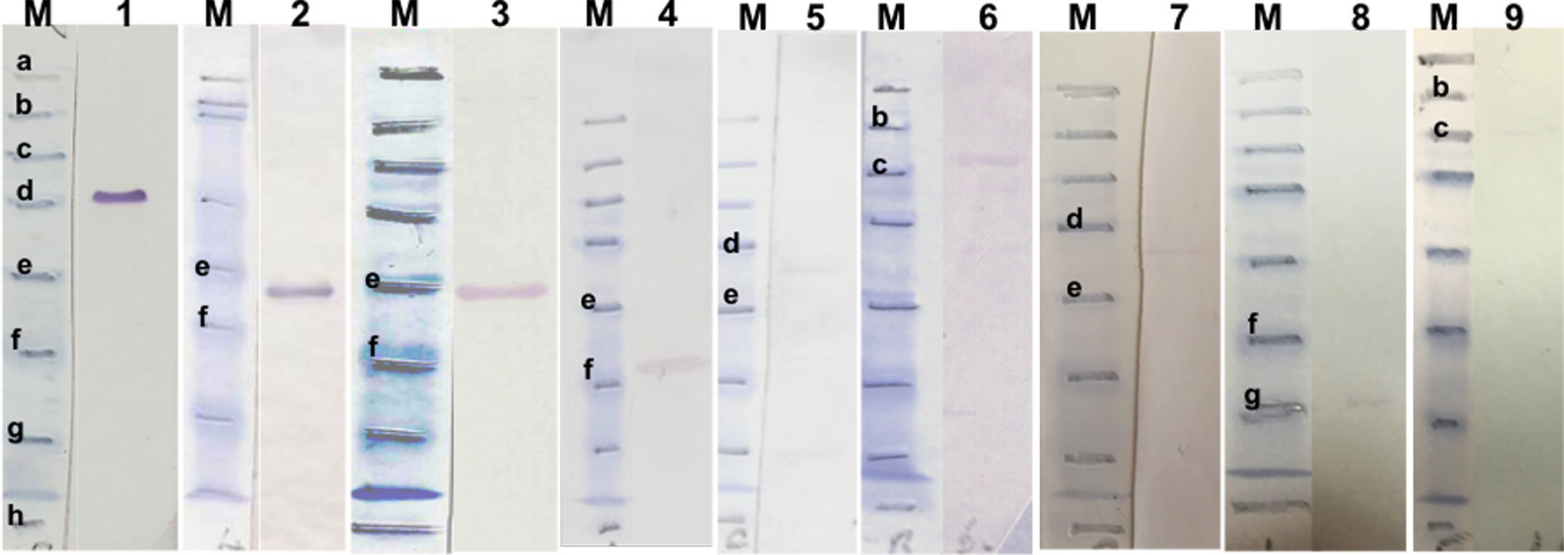
Western blot results of immune sera using recombinant proteins. M, pre-stained SDS-PAGE protein standards (Bio–Rad). Lane 1, recombinant TaSP (rTaSP); 2, rTams; 3, rTA06510; 4, rMero1; 5, rTA13755; 6, rTA11610 (HSP70); 7, rTA03155; 8, rTA20440; 9, rSPAG. Bold, lowercase letters indicate the protein standards between 175–6.5 kDa [a: 175 kDa, b: 83 kDa, c: 62 kDa, d: 47.5 kDa, e: 32.5 kDa, f: 25 kDa, g: 16.5 kDa, h: 6.5 kDa].

Western blot analysis was carried out to quantify the reactivity of recombinant proteins using reference immune serum from experimentally infected animals. This indicated that bioinformatically identified candidates TA06510, TA20440, TA13755 and TA11610 (HSP70) and the previously identified TaSP, Tams1, SPAG and Mero1, were all detected by the reference immune serum (Fig 1). Reactivity, based on the intensity of protein bands detected by immune serum, was found to vary. Compared to the other recombinant proteins, the intensity of TaSP detection was found to be very high. Detection of Tams1 recombinant protein was also high but less than that of TaSP (Fig 1, lines 1 and 2). Among the four candidate antigen genes (*TA06510*, *TA20440*, *TA13755*, *TA11610*), the immune serum showed a moderate reactivity with TA06510 (Fig 1, line 3), however the intensities of the remaining three candidates, TA13755, TA11610 (HSP70) and TA20440 bands were very low (Fig 1, lines 5, 6 and 8). Previously identified recombinant antigen Mero1 was associated with moderately intense band (Fig 1, line 4) while the intensities of TA03155 (Tash1-like), SPAG bands were very low (Fig 1, lines 7 and 9). The immune serum did not show any reactivity with two HSP90 homologue proteins, TA10720 and TA06470 or PQ-rich sub-telomeric proteins, TA16025 and TA17485 (data not shown).

### 3. Western blot analysis of infected cell extracts with immune serum

**Fig 2:**
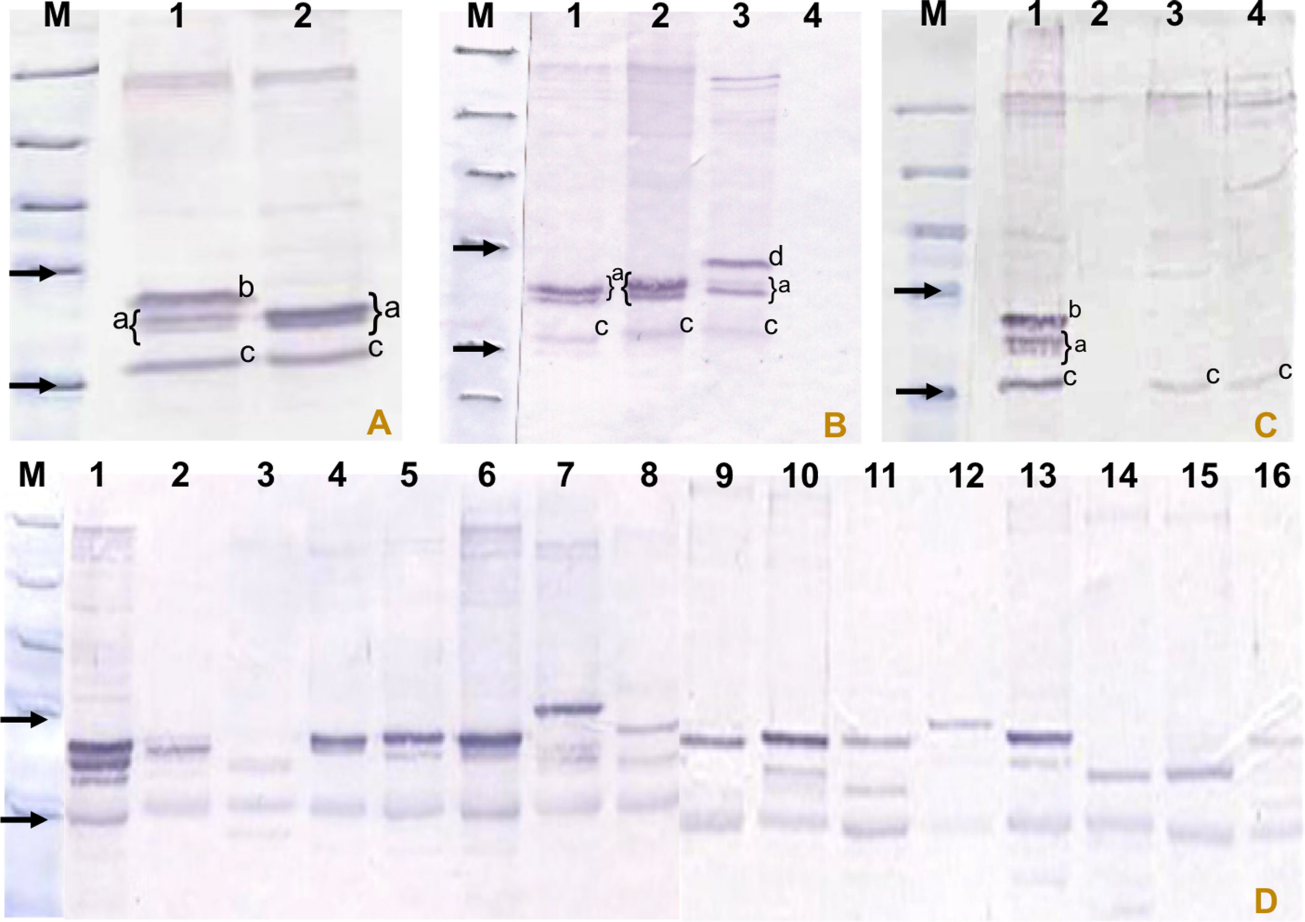
Western blot results of cell lysates from isolates of *T*. *annulata*, TBL and BL20, *T*. *parva* and *T*. *lestoquardi* probed with immune serum. M, pre-stained SDS-PAGE protein standards (Bio–Rad). (A) Lane 1, T.a. A2; 2, T.a. D7. (B) Lane 1, *T*.*a*. D7; 2, *T*.*a*. A46; 3, TBL; 4, BL20. (C) Lane 1, *T*.*a*. A2; 2, BL20; 3, *T*. *parva*; 4, *T*. *lestoquardi*. (D) Lane 1, T.a. A2; 2, T.a. Gharb; 3, T.a. Caceras; 4, *T*.*a*. Ode; 5, *T*.*a*. A46; 6, T.a. J1-1; 7, *T*.*a*. Shambat; 8, *T*.*a*. Umbaneai; 9, *T*.*a*. JED-4; 10, *T*.*a*. BAT-2; 11, *T*.*a*. Pendik; 12, *T*.*a*. Diyarbakır; 13, *T*.*a*. Yenihisar; 14, *T*.*a*. HaciAliObası; 15, *T*.*a*. Akcaova; 16, *T*.*a*. Aydin. Arrows indicate the 32.5 and 47.5 kDa protein standards. ^a^ specific dominant bands in lysates of *Ta*A2, D7 and TBL cells; ^b^ extra specific band in *Ta*A2 lysate; ^c^ common band seen in *T*. *annulata*/*T*. *parva*/*T*. *lestoquardi* lysates; ^d^ additional band seen in BL20 cells infected with *T*. *annulata* (TBL).

Western blot analysis with immune serum obtained from experimentally infected animals identified a number of immunodominant bands in cell lysates of different *T*. *annulata* isolates. Dominant bands showed size polymorphism, varying between 32 to 50 kDa (Fig 2A, 2B and 2D) with the number and intensity of bands varying between isolates. In the TaA2 lysate, four dominant bands were detected (Fig 2A), whereas three dominant bands between 32 and 45 kDa were detected in the D7, TaA46 and TBL20 total cell lysates (Fig 2A and 2B). An additional specific dominant band in the TaA2 lysate was observed at ~43 kDa (Fig 2A). Also, in TBL20 cell lysates an additional protein band with a higher molecular mass (~47.5 kDa) was also detected (Fig 2B). The protein band with the lowest mass (~32 kDa) was detected commonly in *T*. *annulata* A2, *T*. *parva* and *T*. *lestoquardi* cell lysates (Fig 2C) and showed slight size polymorphism across the different isolates of *T*. *annulata* (Fig 2D). Immune serum did not react with total cell lysate of BL20 cells, indicating that the bands detected in infected cell lysates are parasite specific. Also, non-reactivity of pre-immune serum confirmed the specificity of the detected bands (data not shown).

### 4. Immunoabsorption results using TaSP, TA06510 and Tams1 and blocking the detection of antigens in infected cell lysates

**Fig 3:**
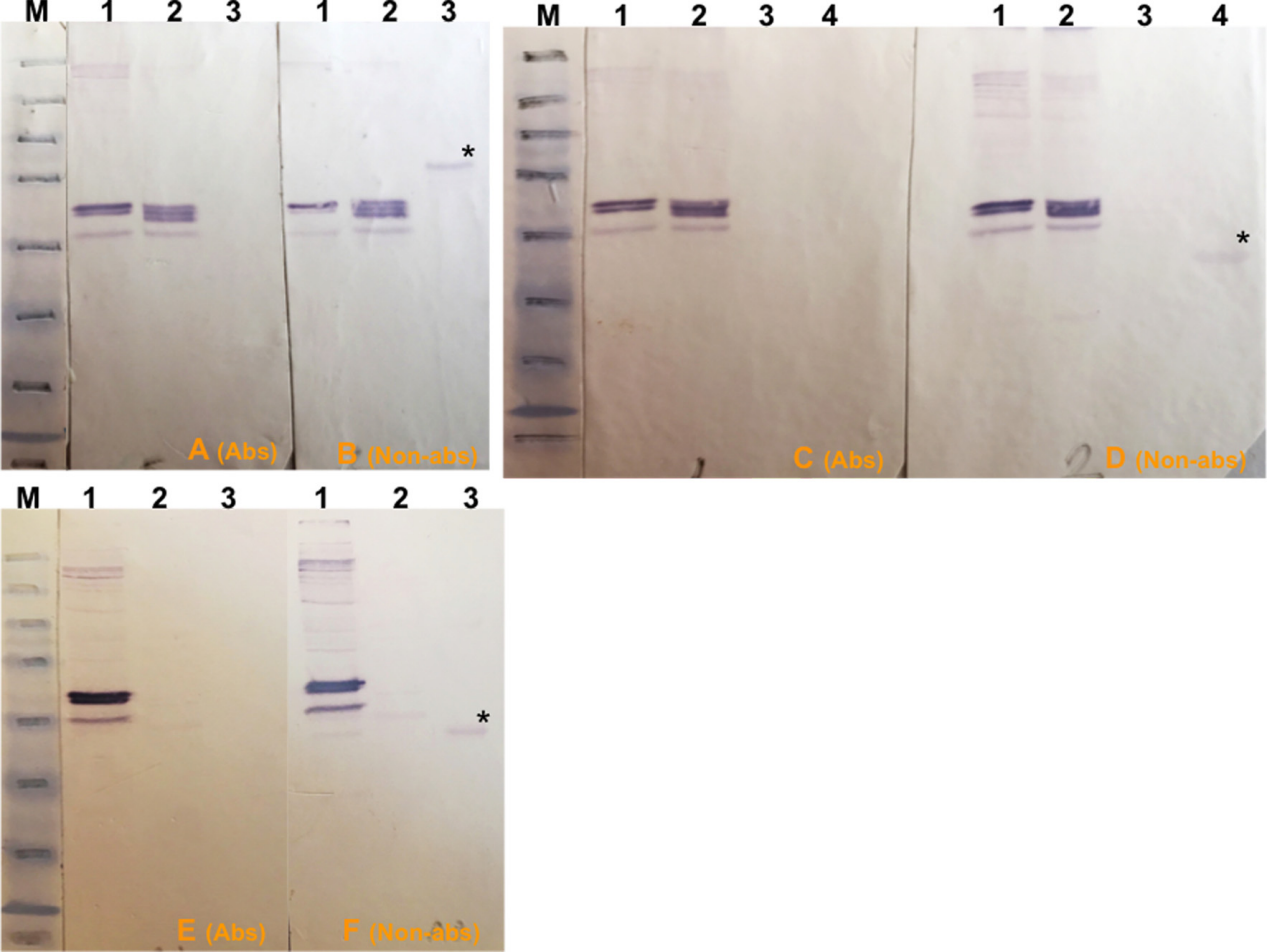
Western blot results of immunoabsorption trials to remove antibodies against rTaSP, rTA06510 and rTams proteins in immune serum. M, pre-stained SDS-PAGE protein standards (Bio–Rad). (A and B) Lane 1, *T*. *annulata*/D7; 2, *T*. *annulata*/A46 clone 4; 3, 1 μg of rTaSP. 1A and 1B filters were probed with immune serum blocked with rTaSP and unblocked immune serum, respectively. (C and D) Lane 1, *T*. *annulata*/D7 extract I; 2, *T*. *annulata*/D7 extract II; 3, BL20; 4, 1 μg of rTA06510. 2A and 2B filters were probed with immune serum blocked with rTA06510 and unblocked immune serum, respectively. (E and F) Lane 1, *T*. *annulata*/D7 extract I; 2, BL20; 3, 1 μg of rTams. 3A and 3B filters were probed with immune serum blocked with rTams and unblocked immune serum, respectively. (*) in filters 1B, 2B and 3B indicates rTaSP, rTA06510 and rTams proteins probed with unblocked immune serum. “Non-abs” and “Abs” indicate the filters that were probed with immune serum blocked with related recombinant proteins and unblocked immune serum, respectively.

An immunoabsorption assay was carried out to determine if immunodominant bands detected by immune serum in infected *T*. *annulata*/Ankara D7 cloned cell line lysates corresponded to any of the highly immunogenic recombinant candidate antigens. Based on initial western blot results, potential antibodies specific for TaSP, Tams1 and TA06510 within the immune serum were adsorbed using the respective recombinant proteins and the adsorbed serum then used in western blots relative to control non-adsorbed immune serum. The efficacy of this process was confirmed, as the treated serum no longer detected recombinant antigens. Western blotting showed that immunodominant bands detected by control untreated serum in *T*. *annulata*/Ankara D7 cell lysates did not correspond to TaSP (Fig 3A and 3B), TA06510 (Fig 3C and 3D) or Tams1 (Fig 3E and 3F). This experiment was repeated using lysates of other *T*. *annulata* isolates and, again, it was shown that none of immunodominant bands corresponded to TaSP, TA06510 or Tams1.

### 5. Immunoprecipitation of infected cell lysate and mass spectrophotometric analysis

An immunoprecipitation assay was then utilised to identify immunodominant bands in *T*. *annulata*/Ankara D7 cloned cell lysates. One band resulted in a MASCOT hit with a hypothetical protein in the *T*. *annulata* genome. This protein is encoded by *TA15705* (see Table D in S1 File), which is a member of a family of genes at a single locus on chromosome 2. This family contains a total of five non-spliced genes each with a conserved signal peptide (1–20 amino acids) at the N-terminus, a low complexity central region and encoding proteins between 175 and 357 amino acids in length (see Fig A in S1 File). GeneDB annotation and membrane topology analysis using TopPred software [65] indicate that each paralogous gene encodes a secreted protein, none of which contain transmembrane domains (see Fig A in S1 File). One member of the family, *TA15685*, was found to possess a FAINT domain within the central region of the encoded protein (see Fig A in S1 File). Details of this gene family are given in Table 1.

### 6. Western blot analysis to detect reactivity of recombinant TA15705 family proteins

**Fig 4:**
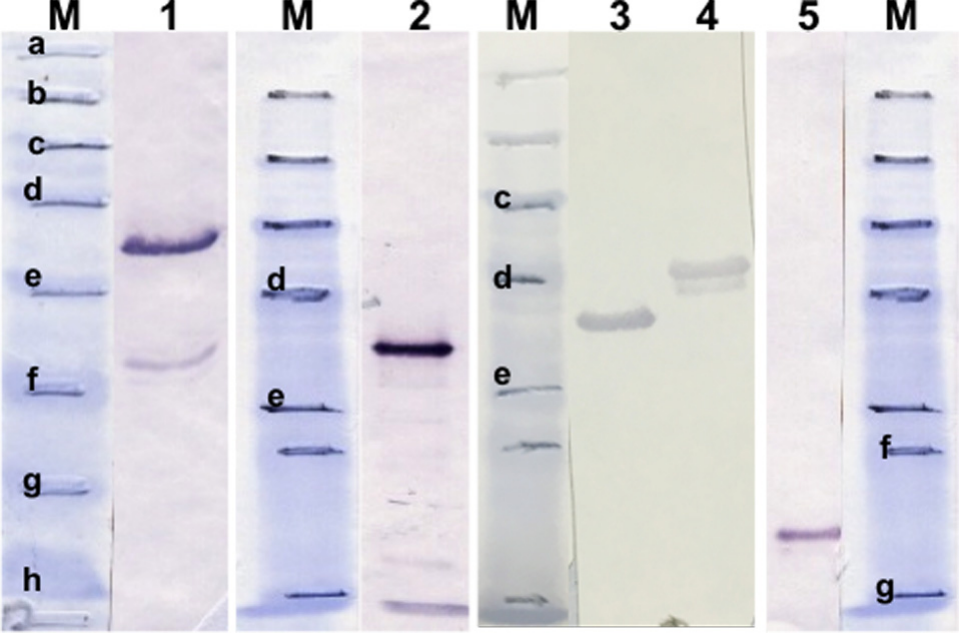
Western blot results of the immune sera using recombinant TA15705 family proteins. M, pre-stained SDS-PAGE protein standards (Bio–Rad). Lane 1, rTA15705; 2, rTA15710; 3, rTA15685; 4, rTA15690; 5, rTA15695. Bold, lowercase letters indicate the protein standards between 175–6.5 kDa [a; 175 kDa, b; 83 kDa, c; 62 kDa, d; 47.5 kDa, e; 32.5 kDa, f; 25 kDa, g; 16.5 kDa, h; 6.5 kDa].

*TA15705* and the other members of this family, i.e. *TA15685*, *TA15690*, *TA15695* and *TA15710*, were cloned and expressed as recombinant proteins. Then, reactivity of these recombinant proteins was quantified by western blot analysis using the reference immune serum. The results indicated that all five paralogues were all antigenic, with TA15705 and TA15710 being the most immunogenic (Fig 4, lines 1 and 2). The band intensities of TA15685 and TA15690 were also high (Fig 4, lines 3 and 4) while that of TA15695 was moderate (Fig 4, line 5).

### 7. Immunoabsorption results using TA15705 family proteins

**Fig 5:**
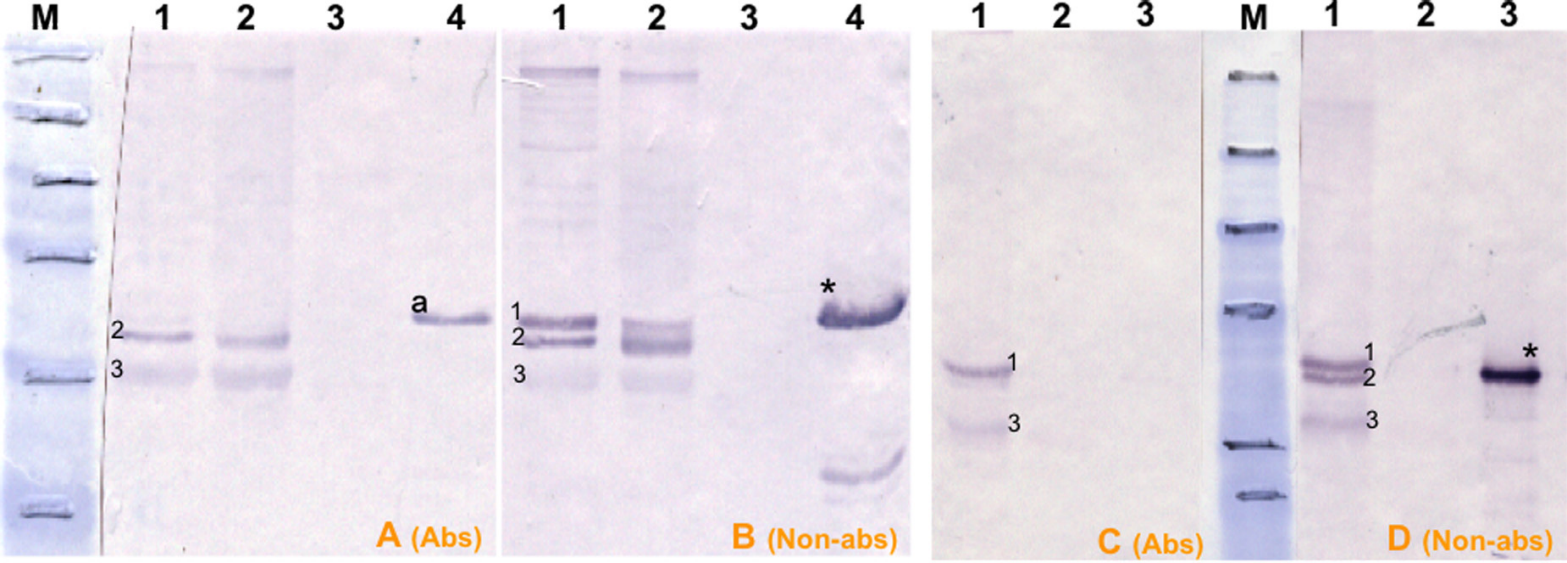
Western blot results of immunoabsorption trials to remove antibodies against rTA15705 and rTA15710 proteins in immune serum. M, pre-stained SDS-PAGE protein standards (Bio–Rad). (A and B) Lanes 1 and 2, *T*. *annulata*/D7 extract I; 3, BL20; 4, 1 μg of rTA15705. A and B filters were probed with immune serum blocked with rTA15705 and unblocked immune serum, respectively. (C and D) Lane 1, *T*. *annulata*/D7 extract I; 2, BL20; 3, 1 μg of rTA15710. C and D filters were probed with immune serum blocked with rTA15710 and unblocked immune serum, respectively. Numbers 1, 2 and 3 in filters B and D indicates immunodominant bands probed with unblocked serum. Numbers 2 and 3 in filter A indicates immunodominant bands probed with immune serum blocked with rTA15705. Numbers 1 and 3 in filter C indicates immunodominant bands probed with immune serum blocked with rTA15710. (*) in filters B, D indicates recombinant TA15705 and TA15710 proteins probed with unblocked immune serum; (^a^) in filter A indicates presence of excess amount of TA15705 specific antibodies reacting with rTA15705. “Non-abs” and “Abs” indicate the filters that were probed with immune serum blocked with related recombinant proteins and unblocked immune serum, respectively.

Immunodominant bands detected by immune serum were further investigated. Anti-TA15705, TA15710, TA15685, TA15690 and TA15695 antibodies in immune serum were adsorbed using the respective recombinant proteins as above. Western blotting using adsorbed and control immune serum showed that immunodominant proteins in D7 cell lysates did not correspond to TA15685, TA15690 or TA15695 proteins, however two bands at approximately 43 and 42 kDa may represent native TA15705 antigen (Fig 5B) and TA15710 (Fig 5D), respectively. Due to either a high level of anti-TA15705-specific antibodies within immune serum or an inefficient absorption by recombinant TA15705 protein, anti-TA15705 antibodies were not completely removed from the immune serum (Fig 5A vs 5B). One possible explanation for inefficient absorption of TA15705 antibodies is variation in the antibody response as a result of underlying allelic variation in the immunising or challenge stabilate with respect to the TA15705 protein.

### 8. ELISA and IFAT results

TA15705 and TA15710 were used to establish an indirect ELISA, which was compared to a previously published TaSP ELISA [21] and IFAT, using a panel of serum samples.

Except in a few individuals, post-challenge antibody titres against TA15705 or TA15710 recombinant proteins showed a rise. ELISA results for these proteins were better in terms of sensitivity than that of the TaSP ELISA. However, stronger detection was observed for the TaSP ELISA with serum from experimentally-infected animals during a primary infection compared to the TA15705 ELISA. This result is supported by data obtained from western blot analysis. ELISA cut-off values for TaSP, TA15705 and TA15710 were chosen arbitrarily to obtain a maximum specificity of at least 95% for each protein. For TA15705 and TA15710 ELISAs, no cross-reaction was detected with any of the sera samples derived from experimental infections with *T*. *parva*, *T*. *buffeli*, *T*. *orientalis*, *B*. *bovis*, *B*. *bigemina*, *A*. *marginale* and *T*. *evansi*. For the TaSP ELISA, cross-reactivity with parasites such as *B*. *bovis* and *Trypanasome* spp. could be negated by increasing the cut-off above 95%. At a chosen cut-off value of 18 PP for the TaSP ELISA, sensitivity and specificity were 89.5% and 98.3%, respectively. The specificity of the TaSP ELISA dramatically reduced below this cut-off value. Using a 21 PP cut-off value for the ELISA based on TA15705 as a recombinant antigen, the specificity of the test was 95.6%; however, the sensitivity was reduced by up to 28.5%. In ELISAs using TA15710 at a cut-off value of 11 PP, sensitivity and specificity were 35% and 98.3%, respectively. Similar to the TaSP ELISA, the specificity of the TA15705 and TA15710 ELISAs reduced to 60% when a lower cut-off value was chosen.

The seroprevalence of *T*. *annulata* in 355 field samples was found to be 50.70% (n = 180), 41.69% (n = 148) and 47.32% (n = 168) with indirect ELISAs based on TaSP, TA15705 and TA15710 recombinant proteins, respectively (Table 2).

**Table 2:** Comparison of IFAT and ELISA results.

Indirect ELISA									
	*TaSP*			TA15705			TA15710		
	Positive	Negative	TOTAL	Positive	Negative	TOTAL	Positive	Negative	TOTAL
IFAT									
Positive	156	77	233*	106	127	233*	137	96	233*
Negative	24	98	122	42	80	122	31	91	122
TOTAL	180*	175	355	148*	207	355	168*	187	355

(*); indicates the total number of positive field samples detected with indirect ELISAs based on TaSP, TA15705 and TA15710 recombinant proteins. The IFAT results given were compared with each of the recombinant protein separately.

**Fig 6:**
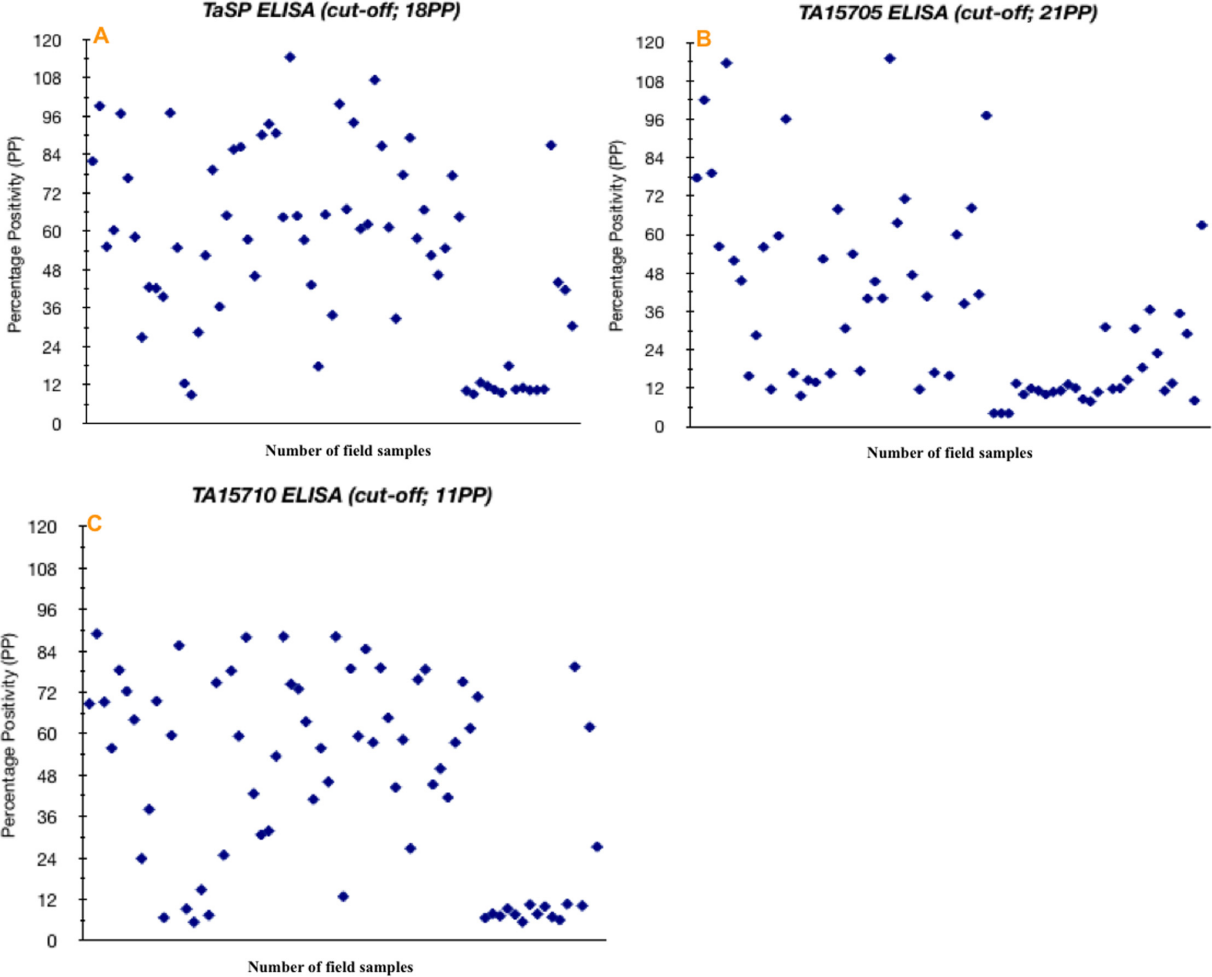
Distribution of percentage positivity (PP) values from field sera samples for TaSP, TA15705 and TA15710. Calculated cut-off values of (A) TaSP, (B) TA15705 and (C) TA15710 indirect ELISAs are 18, 21 and 11, respectively.

The majority (83%) of the PP values in the 180 TaSP ELISA-positive field samples was distributed well above (between 40–100) the established cut-off value; only 35 samples were distributed between PP values of 18 and 36 (Fig 6). In the ELISA using TA15705 as a recombinant antigen, 48% of the PP values were distributed between 21 and 42, while the rest of the positive samples (52%) had PP values between 42 and 139. Within the 168 samples positive with the TA15710 ELISA, 76% and 24% of the PP values were distributed between 22–113 and 11–22, respectively (Fig 6). When the diagnostic efficacy of IFAT was compared with that of the ELISAs, seropositivity increased up to 65.63% (n = 233) in IFATs using macroschizont-infected cells as antigen (Table 2). 174 of 233 IFAT-positive samples were also found to be positive with at least one of the indirect ELISAs (TaSP, TA15705 and TA15710), however 59 out of 233 IFAT-positives were found to be negative in all ELISAs. In addition, while 66 of 122 IFAT-negative animals were negative in all ELISAs, the remaining 56 animals were positive with at least one of the ELISAs. Seroprevalence measured by the TA15710 indirect ELISA (n = 168) was higher than that of TA15705 indirect ELISA (n = 148), yet the TaSP indirect ELISA detected the highest number of positives (n = 180) in field samples. However, PP values of 82 out of 182 TaSP ELISA positive samples were distributed below the calculated thresholds in ELISAs using TA15705 and/or TA15710. Analyses of a selection of field samples also indicated that the TaSP ELISA failed to detect an antibody response in a number of animals (n = 49) that were ELISA-positive using TA15705 and/or TA15710 (Table 2). Thus there is clear variability between the different diagnostic tests with IFAT showing the highest level of sensitivity.

### 9. Sequence polymorphism of *TA15705* and *TA15710* among different isolates

Extensive allelic polymorphism has previously been documented for TA15705 [66], with a hypervariable PQ-rich region identified in the central region of the protein. To assess whether sequence diversity is also a feature of TA15710, allelic sequencing of 11 isolates from different geographical areas was undertaken. Considerable variation was revealed, with a nucleotide diversity (π) of 8.6% across the length of the gene and 84 polymorphic nucleotide sites (k) identified. The ‘Hao’ and ‘Iran’ sequences possessed a premature stop codon in the C-terminal region, resulting in a truncation of 29 amino acid residues compared to the reference sequence. A central polymorphic region was also identified in TA15710 which was enriched for proline (P) and glutamine (Q), however this was less polymorphic than that of TA15705 (see Fig B in S1 File). However, in terms of amino acid diversity, 52 residue positions (42%) in the N-terminal region showed evidence of polymorphism while 44 polymorphic residues (37%) were identified in the C-terminus.

## Discussion

### 1. Comparative analysis of novel and existing recombinant antigens

In an effort to obtain novel improved antigens for use in diagnostic ELISA to detect the carrier state in *T*. *annulata* infected cattle, bioinformatically identified antigen candidates and previously identified antigens (see Table B in S1 File) were expressed as recombinant proteins and tested for reactivity with immune serum by western blotting. The variability in reactivity of recombinant proteins was attributed to differing levels of antigen-specific antibodies generated against each of the native proteins during infection and this may, to an extent, be attributed to allelic polymorphism in the immunising/challenge parasite strains. Among these candidates, two bioinformatically identified TaSP-like hypothetical proteins, TA20440 and TA13755, showed low reactivity, however another bioinformatically identified hypothetical protein, TA06510, was found to be immunogenic and showed a moderate level of detection by immune serum. Bioinformatically identified candidate TA11610 is heat-shock protein 70 (HSP70) and expressed in macroschizont, merozoite and piroplasm stages. Although previous studies in small ruminant theileriosis suggested this protein may be immunogenic [37], western blot results obtained in the present study confirmed low but detectable reactivity against TA11610 (Table 1). This is consistent with another HSP70 of *T*. *annulata*, TA14920, which was previously shown to be unable to generate an immune response [67].

Previously identified merozoite surface antigen, Tams1 has previously been shown to be a suitable immunogenic protein for diagnosis of tropical theileriosis [42]. In the present study, a high level of reactivity against Tams1 was found at 90 days post-infection but reactivity waned with serum obtained during the post-challenge period. Additionally, potential cross-reactivity of recombinant Tams1 with *Babesia bigemina* [45] and some strains of *T*. *parva* [42] would reduce the efficacy of Tams1 as a diagnostic antigen in geographical regions where these pathogens could co-exist. Mero1 is another previously identified polymorphic piroplasm surface antigen, which shows evidence of diversifying selection imposed by the immune system [1]. Reactivity of immune serum against recombinant Mero1 was appreciable, however insufficient for a diagnostic assay.

SPAG and TA03155 (Tash1-like) recombinant proteins were found to have a poor reactivity against immune serum. Theoretically, native SPAG should be recognised early in the course of infection. However, in the absence of repeated sporozoite challenge, antibody titres fade quickly below detectable levels [22] and this was confirmed in the present study. Poor reactivity obtained from a Tash1-like, TA03155, recombinant protein was anticipated due to a high level of conservation among *T*. *annulata* alleles [1].

The immunodominant, *T*. *annulata* surface protein, TaSP, is located on the surface of the macroschizont and possesses a central polymorphic domain [40,41]. TaSP showed strong reactivity with immune serum samples obtained from different infected animals, indicating the existence of high level of immune response against putative B-cell epitopes [40,41]. Compared to the other bioinformatically and previously identified recombinant proteins, a high level of antibody response was also detected in the immune serum used in this study (Table 1) indicating a strong humoral immune response was generated by experimentally-infected animals.

### 2. Detection of native immunodominant antigen by western blot using immune serum

In the present study, western blot analysis, using serum samples obtained from experimentally infected animals with *T*. *annulata* / Ankara sporozoite stabilate, then challenged with *T*. *annulata* / Gharb sporozoite stabilate, identified a number of immunodominant bands in different *T*. *annulata* isolates; a variety of banding patterns was observed. It is highly unlikely that individual animals share an identical immune response and this may be attributed to factors including host and parasite genetic diversity [68]. In the field, a high level of mixed genotype infections within the cattle host have been observed and genotypically distinct parasite populations are known to exist [69–71]. Thus, it is likely that different parasite populations would possess different spectra of polymorphic antigen alleles. Consequently, variation in immunodominant proteins among different *T*. *annulata* isolates may be explained by a combination of variation in the immune responses among individual animals together with genotypic differences between distinct parasite genotypes. Moreover, the level of challenge, the extent of continued exposure and the order in which different genotypes are acquired may also play a role in determining immunodominancy within an individual’s serum.

### 3. Immunodominant bands in infected cell extracts

Based on the western blot results with immune serum, immunodominant divergent antigens are most likely to represent the most sensitive targets in serological assays such as ELISA. To determine whether these antigens represent TaSP or TA06510, an immunoabsorption assay was undertaken. TaSP was believed to be an immunodominant macroschizont antigen based on previous studies [40]. However, our data clearly shows that none of the dominant protein bands detected by immune serum in *T*. *annulata*/Ankara D7 cloned cell lysates corresponded to any of either of these proteins. This indicated that the immunodominant bands of macroschizont-infected cells could be due to recognition of previously unidentified antigens. This hypothesis is supported by demonstration that hypothetical parasite proteins, TA15705 and TA15710, are immunodominant in the system under study. Previously identified as an immunodominant T-cell antigen Ta9, TA15705 [72] was found in the present study to also be strongly detected by the humoral response, indicating that TA15705 has both T- and B-cell epitopes. It is unknown whether TA15710 also has T-cell epitopes and this requires further investigation.

The presence of a high level of antibody response against TA15705 was found post-challenge but not during primary infection. However, this difference is not currently clearly understood. It may be hypothesised that the elevated antibody response post-challenge may be related to lysis of infected cells by a CTL response in immune animals resulting in the release of Ta9 present in the host cytoplasm and thus its exposure to the humoral immune system.

### 4. ELISA vs IFAT results

During the course of infection, the immune system of cattle is exposed to a variety of antigens expressed during different life-cycle stages of the parasite [29,73,74]. To date, a number of antigens specific to different bovine life-cycle stages have been used for ELISA [33–35,42,67], however they have been considered unsuitable for ELISA-based robust diagnosis of infection due to sensitivity and/or specificity issues. It is currently unclear which antigen or antigens represent the most sensitive and specific ELISA for the detection of the carrier state of *T*. *annulata*. It is possible that novel highly immunogenic antigens can be utilised to produce a highly sensitive ELISA assay. The highly immunogenic *T*. *annulata* schizont surface protein, TaSP, has a high level of polymorphism within and between parasite isolates [46]. Recombinant TaSP has extensively been used in ELISA-based diagnosis of *T*. *annulata* [20,21,36,41,44,75,76]. Based on the hypothesis that other antigens may provide a superior target, two immunodominant recombinant proteins, TA15705 and TA15710 identified in this study, were used to establish indirect ELISAs, which were compared to the established TaSP ELISA [21]. The diagnostic efficacy of each indirect ELISA was tested in comparison to IFAT. Despite its extensive use in the field, the TaSP ELISAs is known to have issues with sensitivity and specificity [20,75]. In previous studies, the sensitivity of TaSP ELISAs was kept at up to 99.1%, but specificity of the test at this level was insufficiently high (90.47%) to exclude potential cross-reactivity. In the present study, optimal PP values that gave highest sensitivity without reducing the specificity were picked for each of the ELISAs using recombinant TaSP, TA15705 and TA15710. As a result, no cross-reaction was observed with any of the control sera for indirect ELISAs using TA15705 or TA15710. For TaSP, the chosen PP value eliminated cross-reactivity with *B*. *bovis* and *Trypanasome* spp. [20].

The seroprevalence of *T*. *annulata* determined in 355 field samples varied between indirect ELISAs using TaSP, TA15705 and TA15710 recombinant proteins. In comparison to TaSP, TA15705 and TA15710 ELISAs, a greater level of seropositivity was obtained for IFAT (Table 2). However, IFAT can cross-react with other *Theileria* species [77]. In the present study, the TaSP indirect ELISA detected the highest number of positives in the field samples, however it failed to detect an antibody response in a number of animals that were ELISA-positive using TA15705 and/or TA15710 (Table 2). This raised the question of whether TaSP overestimates or underestimates exposure to *T*. *annulata*. The data obtained from established indirect ELISAs and western blots using recombinant TA15705, TA15710 and TaSP antigens indicate that variable results across tests may be due to differential immune responses among individual infected animals and this may be driven, in part, by allelic sequence polymorphism across different parasite stocks. Also, TaSP has a high level of allelic diversity within the central polymorphic region and displays both size and amino acid sequence polymorphism, within single parasite isolates and between isolates from different geographical regions [46]. Thus polymorphic TaSP alleles may result in the generation of varying immune responses among individual animals. It is known that CD8^+^ cytotoxic T cell responses against TA15705 (Ta9) differ between heterologous stocks of *T*. *annulata* [72] and this has been related to allelic variation in the protein. The gene sequence of TA15705 shows length polymorphism and indels within the PQ-rich central region of the predicted polypeptide (see Fig B in S1 File). Another immunodominant member of this family, TA15710 shows slightly less polymorphism (see Fig B in S1 File) and detected a slightly greater number of positive serum samples. Similar to TaSP, variable detection of infected animals by TA15705 and TA15710 ELISAs may also be a result of antigen polymorphism and divergent immune recognition. Alternatively, this may be due to reactivity against peptide motifs shared between other proteins [78]. These results may indicate that no single antigen can generate an ELISA test optimal for detection of the carrier state in *T*. *annulata*. Variable detection could be circumvented by use of a species-specific conserved antigen, but none have been identified to date that also provide the requisite sensitivity. One possibility for future studies would be to undertake a more in-depth analysis of antigenic diversity for chosen candidates and utilise this to generate recombinant antigen representing divergent alleles for development of a diagnostic ELISA.

## Conclusions

The present results indicate that genome-mining is a useful technique for identifying immunodominant antigen genes that provide candidates for use in an ELISA capable of detecting *T*. *annulata*-infected carriers. Unfortunately, the results also indicate that antigen diversity is associated with immunodominant antigens and that none of the recombinant proteins tested in the present study out-performed TaSP as an ELISA target. Further work is required to identify novel diagnostic targets or a combination thereof in order to improve the sensitivity and specificity of *T*. *annulata* ELISA assays.

## Supporting Information

S1 File Fig A, Chromosomal location and features of *TA15705* paralogue family genes. Fig B, Comparison of allelic polymorphism within *TA15705* and *TA15710* amino acid sequences. Table A, Origin and nature of parasite stocks. Table B, Oligonucleotide primers and expression vectors used to express recombinant proteins. Table C, Antigen, serum and conjugate dilutions used for indirect ELISA. Table D, Peptide hit data of peptide sequences generated by a search through Mascot against all sequences in NCBI.
(DOC)
