# Supplementary material for: Identification and Analysis of Immunodominant Antigens for ELISA-Based Detection of Theileria annulata
Source: PLoS One. 2016 Jun 7;11(6):e0156645. doi: 10.1371/journal.pone.0156645 (PMC4896419; doi:10.1371/journal.pone.0156645)
Supplement: S1 File — Fig A, Chromosomal location and features of TA15705 paralogue family genes. Fig B, Comparison of allelic polymorphism within TA15705 and TA15710 amino acid sequences. Table A, Origin and nature of parasite stocks. Table B, Oligonucleotide primers and expression vectors used to express recombinant proteins. Table C, Antigen, serum and conjugate dilutions used for indirect ELISA. Table D, Peptide hit data of peptide sequences generated by a search through Mascot against all sequences in NCBI. (DOC) [file pone.0156645.s001.doc]

**Supplementary Figures and Tables in S1 File**

**Figure A.** **Chromosomal location and features of *TA15705* paralogue family genes**


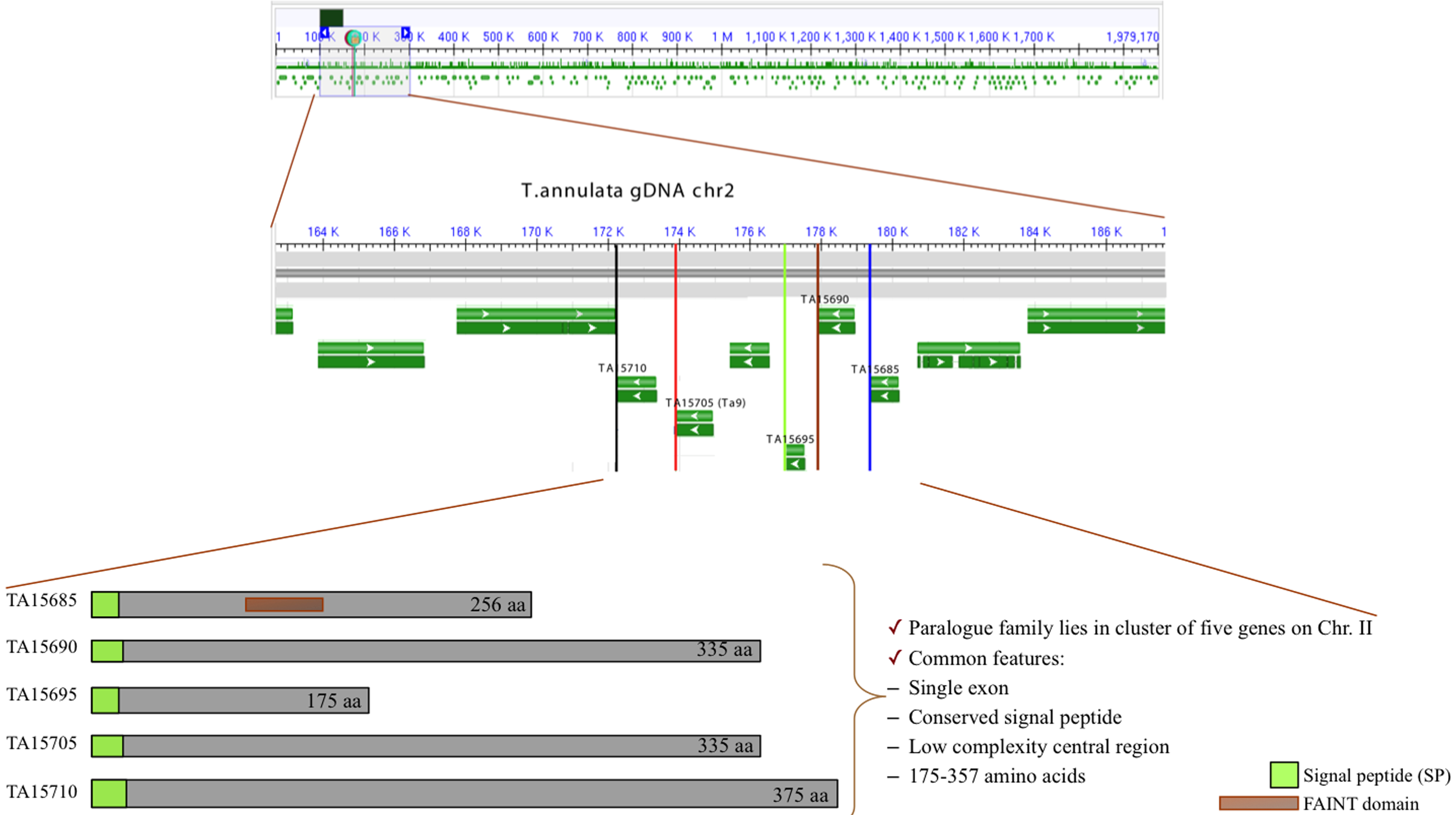


**Figure B. Comparison of allelic polymorphism within *TA15705* and *TA15710* amino acid sequences**


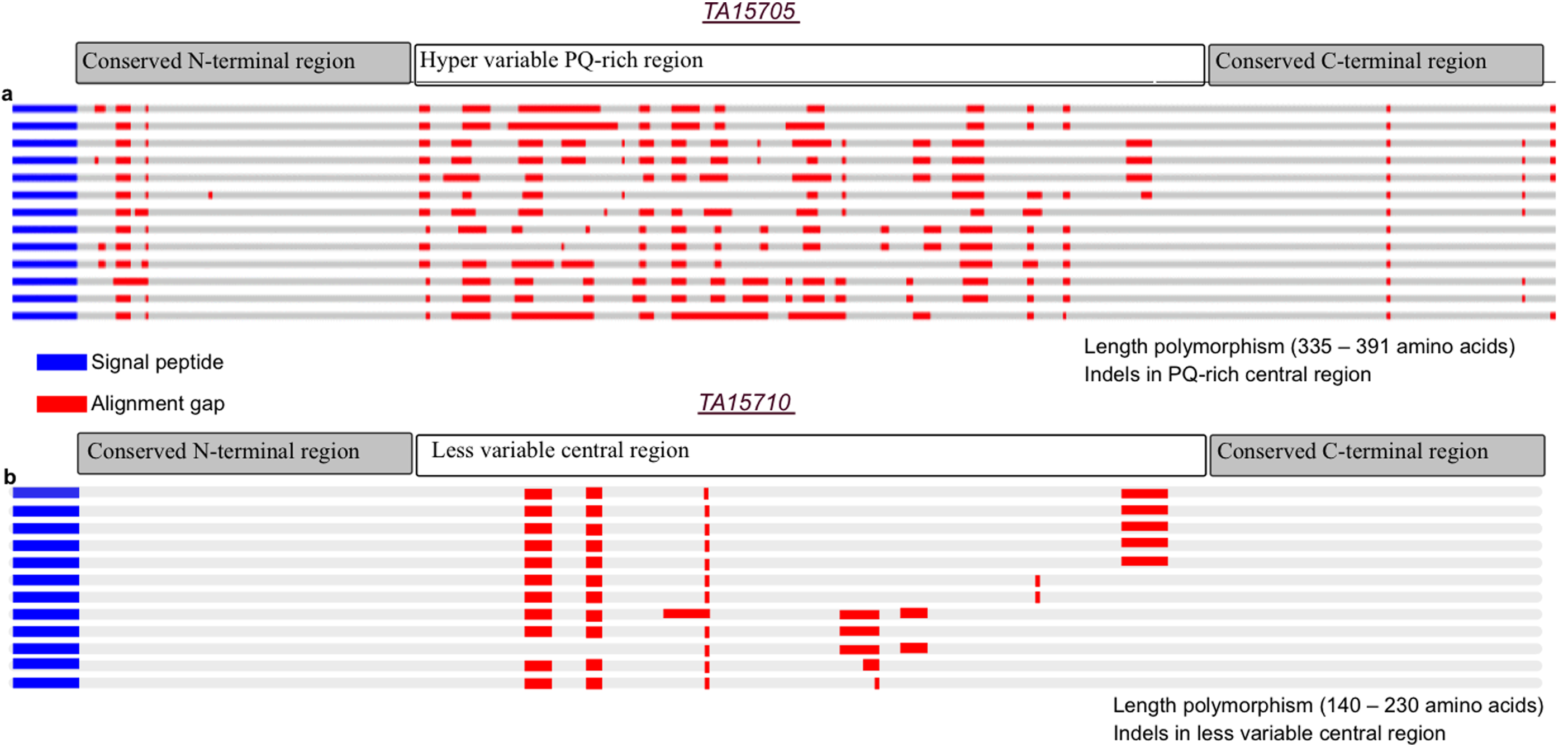


Comparison of allelic polymorphism within *TA15705* and *TA15710* amino acid sequences. (^a^ ) indicates distinct *T. annulata* alleles and (^b^) indicates alleles identified among different isolates of *T. annulata* (*T. annulata*/Akcaova; *T. annulata*/Dalama; *T. annulata*/Kocarlı*; T. annulata*/Hacı Ali Obası; *T. annulata*/YeniHisar; *T. annulata*/Diyarbakır; *T. annulata*/Pendik; *T. annulata*/Morocco, Gharb; *T. annulata* / Iran; *T. annulata*/India, Ode; *T. annulata*/Israel, Tova; *T. annulata*/Tunisia, JED-4).

**Table A. Origin and nature of parasite stocks**

| Genus and species | Stock origin |  | Material | Reference |
| --- | --- | --- | --- | --- |
|  | Country | Location or name |  |  |
| *Theileria annulata* | Turkey | Ankara (D7) | cell line | Shiels et al. 1992 |
| *" "* | Turkey | Ankara (A46) | cell line | " " |
| *" "* | Turkey | Ankara (A2) | cell line | " " |
| *" "* | Turkey | Akçaova | cell line | Adnan Menderes Univ., Fac. of Med. Vet., Dept. of Parasitology |
| *" "* | Turkey | HacıAli Obası | cell line | " " |
| *" "* | Turkey | Yenihisar | cell line | " " |
| *" "* | Turkey | Aydın | cell line | " " |
| *" "* | Turkey | Pendik | cell line | Özkoc and Papino, 1981 |
| *" "* | Turkey | Diyarbakır | cell line | Weir, 2006 |
| *" "* | Tunisia | JED–4 | cell line | Ben Miled et al. 1994 |
| *" "* | Tunisia | BAT2 | cell line | " " |
| *" "* | Tunisia | J1-1 | cell line | " " |
| *" "* | Sudan | Umbaneai | cell line | Shiels et al. 1986 |
| *" "* | Morocco | Gharb | cell line | Ouhelli et al. 1989 |
| *" "* | India | Ode | cell line | Baylis et al. 1992 |
| *" "* | Sudan | Shambat | cell line | Shiels et al. 1986 |
| *" "* | Spain | Caceras | cell line | De Kok et al. 1983 |
| *Theileria parva* | Kenya | Muguga | cell line | Oura et al. 2003 |
| *Theileria lestoquardi* | Iran | Lahr | cell line | Kıvrar et al. 1998 |
| BL20 | uninfected bovine lymphoma cells |  | cell line | Shiels et al. 1986 |
| TBL | BL20 cells infected with *T. annulata* / D7 sporozoites |  | cell line | Shiels et al. 1986 |

**Table B. Oligonucleotide primers and expression vectors used to express recombinant proteins**

| GeneDB accession no. | Primer sequences (5'-3')^a,b^ | M_r_ | M_r_ with His-tag | Amplicon length (bp) | Protein expression (vector/strain/induction substance)^c^ |
| --- | --- | --- | --- | --- | --- |
| *TA06510* | F;GATATTGAGGAGGATCTACGTAGA  R;ACTTGATAATCTGTATGGGATTTT | 34.6 | 35.4 | 804 | pDEST17/BL21-AI/L-arabinose |
| *TA20440* | F;GAAAATTTTAACAATAAAAGGTGT  R;TTGAGACTCCATGTCTCTCATGGT | 12 | 12.8 | 318 | pDEST17/BL21-AI/L-arabinose |
| *TA13755* | F;GAACTTTTACCACACACCGGTGAT  R;GGGTTTTTCAAATCCTGAAGGTAC | 16 | 16.8 | 442 | pDEST17/BL21-AI/L-arabinose |
| *TA15705* | F;ATGGATTCTTCTGATTCTGATGAA  R;ATCCTTTTCTTCCCATGGTTTGGC | 34.9 | 35.7 | 951 | pDEST17/BL21-AI/L-arabinose |
| *TA15710* | F;ATGGATCCTGATGGATCTGAACCT  R;TTATTGTTTTTCTATATCACGTTT | 37.9 | 38.7 | 1020 | pDEST17/BL21-AI/L-arabinose |
| *TA15685* | F;ATGGATCCTGAAGATGGATCTGAG  R;TTATCTTGGTGTTACTTGTACCCA | 28 | 28.8 | 714 | pDEST17/BL21-AI/L-arabinose |
| *TA15690* | F;ATGGATCCTGATGGAGCTGAACCT  R;TCAAGAAAGTGCTTGTCTAATAAA | 35.9 | 36.7 | 954 | pDEST17/BL21-AI/L-arabinose |
| *TA15695* | F;ATGGATCCTGATGGAGCTGATGG  R;CTAATTCTTCTCTTCCCATGGTTT | 19 | 19.8 | 474 | pDEST17/BL21-AI/L-arabinose |
| *TA11610* | F;ACAGGACCAGCTATTGGAATTGAC  R;ATCCACCTCCTCTACAGTTGGGCC | 71 | 71.8 | 1935 | pDEST17/BL21-AI/L-arabinose |
| *TA17315* | F;GGTCCATTTCTTCCTTTAGATCGAC  R;CCTTCGGGCGCTTATCATGATCGGA | 19.1 | 19.9 | 516 | pDEST17/BL21-AI/L-arabinose |
| *TA17050** | expressed from available glycerol stocks | 32.3 | 33.1 | 843 | pQE-60/M15 (pREP4)/IPTG |
| *TA03755** | expressed from available glycerol stocks | 90.9 | 91.7 | 2694 | pQE-60/M15 (pREP4)/IPTG |
| *TA13810** | expressed from available glycerol stocks | 22.4 | 23.2 | 627 | pDEST17/BL21-AI/L-arabinose |
| *TA03155** | expressed from available glycerol stocks | 32.8 | 33.6 | 831 | pQE-60/M15 (pREP4)/IPTG |
| *TA10720** | expressed from available glycerol stocks | 104.2 | 105 | 2739 | pQE-60/M15 (pREP4)/IPTG |
| *TA06470** | expressed from available glycerol stocks | 113.2 | 114 | 2901 | pQE-60/M15 (pREP4)/IPTG |
| *TA16025** | expressed from available glycerol stocks | 64.5 | ?? | ?? | pDEST17/BL21-AI/L-arabinose |
| *TA17485** | expressed from available glycerol stocks | 47.5 | ?? | ?? | pDEST17/BL21-AI/L-arabinose |

^a^ primers were used with forward attB (GGGACAAGTTTGTACAAAAAAGCAGGCTTC-) and reverse attB adapters (GGGGACCACTTTGTACAAGAAAGCTGGGTCCTA-) at 5' end of each primer sequence.

^b^ primers were used for gene amplification by traditional PCR from gDNA.

^c^ recombinant proteins were expressed with a 6xHis tag.

* recombinant proteins expressed from available glycerol stocks held by Institute of BAHCM, University of Glasgow.

**Table C. Antigen, serum and conjugate dilutions used for indirect ELISA**

|  | *TaSP* | *TA15705* | *TA15710* |
| --- | --- | --- | --- |
| Recombinant Ag (1 µg) | 1/320 | 1/80 | 1/160 |
| Serum | 1/160 | 1/120 | 1/320 |
| Conjugate (HRPO)* | 1/15000 | 1/15000 | 1/15000 |

* rabbit anti-bovine IgG horseradish peroxidase-labelled conjugate (HRPO) developed in rabbit (Sigma; A-5295)

**Table D.** **Peptide hit data of peptide sequences generated by a search through Mascot against all sequences in NCBI**

| **Accession code** | **Annotation** |
| --- | --- |
| [gi|27807377](http://fun-gen1.ibls.gla.ac.uk/mascot/cgi/master_results.pl?file=../data/20080303/F051489.dat#Hit1) | glutamic-oxaloacetic transaminase 2, mitochondrial (aspartate aminotransferase 2) [Bos taurus] |
| [gi|109659291](http://fun-gen1.ibls.gla.ac.uk/mascot/cgi/master_results.pl?file=../data/20080303/F051489.dat#Hit2) | Acetyl-Coenzyme A acetyltransferase 2 [Bos taurus] |
| [gi|223046](http://fun-gen1.ibls.gla.ac.uk/mascot/cgi/master_results.pl?file=../data/20080303/F051489.dat#Hit3) | aminotransferase,Asp |
| [gi|78369456](http://fun-gen1.ibls.gla.ac.uk/mascot/cgi/master_results.pl?file=../data/20080303/F051489.dat#Hit21) | nucleophosmin (nucleolar phosphoprotein B23, numatrin) [Bos taurus] |
| [gi|77735757](http://fun-gen1.ibls.gla.ac.uk/mascot/cgi/master_results.pl?file=../data/20080303/F051489.dat#Hit40) | acyl-Coenzyme A dehydrogenase, C-2 to C-3 short chain [Bos taurus] |
| [gi|115497482](http://fun-gen1.ibls.gla.ac.uk/mascot/cgi/master_results.pl?file=../data/20080303/F051489.dat#Hit41) | zinc binding alcohol dehydrogenase, domain containing 1 [Bos taurus] |
| [gi|84994198](http://fun-gen1.ibls.gla.ac.uk/mascot/cgi/master_results.pl?file=../data/20080303/F051489.dat#Hit47) | **hypothetical protein *TA15705* [*Theileria annulata* Ankara]** |
